# PREAL: prediction of allergenic protein by maximum Relevance Minimum Redundancy (mRMR) feature selection

**DOI:** 10.1186/1752-0509-7-S5-S9

**Published:** 2013-12-09

**Authors:** Jing Wang, Dabing Zhang, Jing Li

**Affiliations:** 1 Bor Luh Food Safety Center, National Center for Molecular Characterization of Genetically Modified Organisms, State Key Laboratory of Hybrid Rice, School of Life Science and Biotechnology, Shanghai Jiao Tong University, 800 Dongchuan Rd, Shanghai 200240, People's Republic of China; 2 Department of Bioinformatics & Biostatistics, School of Life Science and Biotechnology, Shanghai Jiao Tong University, China; 3 Shanghai Center for Bioinformation Technology, China

## Abstract

**Background:**

Assessment of potential allergenicity of protein is necessary whenever transgenic proteins are introduced into the food chain. Bioinformatics approaches in allergen prediction have evolved appreciably in recent years to increase sophistication and performance. However, what are the critical features for protein's allergenicity have been not fully investigated yet.

**Results:**

We presented a more comprehensive model in 128 features space for allergenic proteins prediction by integrating various properties of proteins, such as biochemical and physicochemical properties, sequential features and subcellular locations. The overall accuracy in the cross-validation reached 93.42% to 100% with our new method. Maximum Relevance Minimum Redundancy (mRMR) method and Incremental Feature Selection (IFS) procedure were applied to obtain which features are essential for allergenicity. Results of the performance comparisons showed the superior of our method to the existing methods used widely. More importantly, it was observed that the features of subcellular locations and amino acid composition played major roles in determining the allergenicity of proteins, particularly extracellular/cell surface and vacuole of the subcellular locations for wheat and soybean. To facilitate the allergen prediction, we implemented our computational method in a web application, which can be available at http://gmobl.sjtu.edu.cn/PREAL/index.php.

**Conclusions:**

Our new approach could improve the accuracy of allergen prediction. And the findings may provide novel insights for the mechanism of allergies.

## Background

Allergens are something that can induce type-I hypersensitivity reaction in atopic individuals mediated by Immunoglobulin E (IgE) responses [1-4], which are seriously harmful to human health. For instance, allergenic proteins in food and other hypersensitivity reactions are major causes of chronic ill health in affluent industrial nations, mostly against milk, eggs, peanuts, soy, or wheat, affecting up to 8% of infants and young children [5-7]. Moreover, the introduction of genetically modified foods and new modified proteins is increasing the risk of food allergy in susceptible individuals as well [8,9]. Consequently, assessing the potential allergenicity of proteins is essential to prevent the inadvertent generation of new allergenic food by agricultural biotechnology.

In 2001, the World Health Organization (WHO) and Food and Agriculture Organization (FAO) proposed guidelines to assess the potential allergencity of a protein, an important part of which is to use bioinformatic methods to determine whether the primary structure (amino acid sequence) of a given protein is sufficiently similar to sequences of known allergenic proteins [10,11]. In FAO/WHO rules, a protein is identified as a putative allergen if it has at least six contiguous amino acids matched exactly (rule 1) or a minimum of 35% sequence similarity over a window of 80 amino acids (rule 2) when compared with known allergens. Some researches have shown that the bioinformatic rules of FAO/WHO produced many false positives for allergen prediction [12-19]. Since then, a number of other computational prediction methods based on the protein structure or sequence similarity comparing with known allergens have been reported [18,20-26]. For example, a new approach brought an increase of the precision from 37.6% to 94.8% by identifying motifs from known allergen in 2003 [18]. Statistical learning method SVM (support vector machine) was used for predicting allergens since 2006, and the input features of most SVM-based prediction approaches were compose of either amino acid composition or pair-wise sequence similarity score with known allergens' [20-24,27]. Furthermore, using identifying epitope, allergen representative peptides or family featured peptides were also applied in the allergen prediction [20,25,26]. But the usage of these two methods was limited because very few epitopes and allergen representative peptides have been known until now.

In our previous study, it's observed that, although FAO/WHO criteria have a higher sensitivity and the motif-based approach may give a graph view on the key allergenic motif, we found that the SVM-based method is superior to the others in the accuracy of allergen prediction and processing time [28]. As described as above, a variety of bioinformatic methods for predicting allergen have been reported, most of these approaches depend upon the similarity of protein sequence or primary sequential properties between query protein and the known allergens only. Here, besides protein sequential features, we developed an improved model for identifying potential protein allergenicity using 128 features in terms of their biochemical, physicochemical, subcellular locations. And then, all features were ranked using mRMR (maximum relevance & minimum redundancy) method and an optimal model was rebuilt and evaluated with ten-fold cross validations. At last, we presented a web-based application with a friendly interface that allows users submit individual or batch prediction with query protein or protein list using our new method.

## Methods

### Datasets

1176 distinct allergen proteins were collected from Swiss-Prot Allergen Index, IUIS Allergen Nomenclature, SDAP [26] and ADFS [29], and were used as the positive dataset. To build a reliable negative dataset, we integrated the previously reported methods[13,18,22], and the following processing was done: (1) 522,019 protein entries were downloaded from Swiss-Prot (Swiss-Prot Release 2010_11 of 02-Nov-10); (2) the entries were removed, of which sequence identities > = 30% with any known allergen; (3) all sequences less than 50 amino acid were also discarded; (4) the same number of the negative samples were selected randomly from the remaining subjects in the following cross-validations of the evaluation.

### Software

NCBI-BLAST (version 2.2.23) was used to find the similarity between sequences [30]. SSpro/ACCpro 4.03 [31,32], for predicting secondary structure and solvent accessibility of protein, were obtained from http://download.igb.uci.edu/. In order to access a protein as an allergen or non-allergen, SVM method was implemented using LIBSVM software v3.0 [33], from http://www.csie.ntu.edu.tw/~cjlin/libsvm/. The mRMR program [34], from http://penglab.janelia.org/proj/mRMR/, was acquired for feature ranging and selection. A Perl script was written for protein features extraction and allergenicity prediction. ClustalX2 and Muscle was used for multiple sequence alignments with the default parameters [35,36]. The NJ (Neighbour-Joining) tree was constructed with the aligned protein sequences using MEGA (version 5) with the following parameters: poisson correction, pairwise deletion, and bootstrap (1,000 replicates; random seed) [37].

### Feature vector construction

### (1) Features of biochemistry and physicochemistry

The following six kinds of biochemical and physicochemical properties were extracted from a given protein sequence: (1) amino acid composition (AAC), (2) molecular weight (MW), (3) hydrophobicity, (4) polarizability, (5) normalized van der Waals volume (NWV), and (6) polarity.

AAC is the fraction of each amino acid in a protein [20]. The fraction of all 20 natural amino acids was calculated using the Eq. (1).

(1) $$\text{Fraction of amino acid }i=\frac{\text{total number of amino acids}\left(i\right)}{\text{total number of amino acids in protein}},$$

where  ican be any amino acid.

The molecular weight was considered in this study since some researches showed that it's related with allergen identification [38-42]. Except for AAC and MW that reflect global feature of a protein, of the above six types of properties, the construction of all the other four types of biochemical and physicochemical properties, which is related with a single amino acid in a given protein sequence was adopted from the report of Huang et al. [43]. Each of these local types of properties can be classified into three categories. For instance, an amino acid can be grouped as: polar, neutral or hydrophobic for the hydrophobicity. Similarly, the classifications of polarizability, NWV and polarity were also summarized in Table 1[44-46]. And then, in term of each type of property above, the 20 elements of original protein sequence can be recoded using the corresponding three local features such as P (polar), N (neutral) and H (hydrophobic). At last, with method developed by Huang et al. [43], the coded sequence can be integrated into the corresponding global features: C (composition), T (transition) and D (distribution). C refers to the global composition of each of the three groups (3 elements), while T is defined as the proportion of transformation of each pair letters on the total changes along the entire coded sequence (3 elements), and D expresses the distribution pattern of the code letters which is measured by the position of the first, 25%, 50%, 75%, and 100% of each of the three letters along the sequence (5*3 = 15 elements). Therefore the properties which classified into three categories would generate 21 features each (3+3+15 = 21).

**Table 1:** The classification of protein properties

Property type	Category	Amino acid
**Hydrophobicity**	Polar	R, K, E, D, Q, N
	Neutral	G, A, S, T, P, H, Y
	Hydrophobic	C, V, L, I,M, F,W

**Polarizability**	0-0.108	G, A, S, D, T
	0.128-0.186	C, P, N, V, E, Q, I, L
	0.219-0.409	K, M, H, F, R, Y, W

**NVV^a^**	0-2.78	G, A, S, C, T, P, D
	2.95-4.0	N, V, E, Q, I, L
	4.43-8.08	M, H, K, F, R, Y, W

**Polarity**	4.9-6.2	L, I, F, W, C, M, V, Y
	8.0-9.2	P, A, T, G, S
	10.4-13.0	H, Q, R, K, N, E, D

**SSP^b^**	Helix	Predicted by SSpro [31]
	Strand	
	Coil	

**Solvent**	Buried	Predicted by ACCpro [32]
	Exposed	

^a^normalized van der Waals volume; ^b^secondary structure propensity.

### (2) Subcellular location description of proteins

The protein's subcellular location information was also incorporated in input features for SVM, because it is closely correlated with the function of a protein [47,48]. There were 22 subcellular locations for eukaryotic proteins collect from UniProt [49], therefore, we represented the subcellular location features by a 22-dimensional vector $SL=\left(sl_{1},sl_{2},sl_{3},\cdots \;,sl_{22}\right)$, where $sl_{i}=1$ refers that the query protein is located at the  i -th subcellular location site. Conversely, $sl_{i}=0$ refers that the query protein is not found at the  i -th subcellular location site [43]. However, proteins have subcellular location annotations are in the minority. In order to solve this issue, we predicted the localization information for those without annotation based on the sequence similarity with location-known proteins. Upon the sequence similarity evaluated by BLAST [30], the query protein was considered to have the same subcellular locations with a location-known protein if the BLAST score was greater than 120 between them [43].

### (3) Feature space

As mentioned above, hydrophobicity, polarizability, NWV and polarity generated 21 elements each. And there were 20 elements for AAC, 1 element for MW and 22 for subcellular locations. In addition, the length of protein was also counted as a component. Therefore, the total feature space to represent a protein sample contained (21*4+20+1+22+1) = 128 components, as listed in Additional file 1 for the details. Consequently, a protein sample can be formulated as a vector in a 128-D (dimensional) space; i.e.,

(2) $$V={\left[v_{1},v_{2},v_{3},\cdot \cdot \cdot ,v_{j},\cdot \cdot \cdot ,v_{128}\right]}^{\text{T}\;}$$

where $v_{j}^{}$ is the  j-th ( j = 1,2,...,128) component of the protein.

To enhance the accuracy of SVM, each of the 128 features in Eq.2 was scaled by Eq.3.

(3) $$v_{j}=\left(v_{j}-\mu_{j}\right)/)\sigma_{j}\left(j=1,2,\cdots \;,128\right)$$

where $\mu_{j}$is the mean, and $\sigma_{j}$is the standard deviation of the  j -th component over all protein samples.

### Feature selection

### (1) mRMR method

mRMR method was developed to rank each feature according to its relevance to the target and redundancy with other features [34]. The program of mRMR was downloaded from http://penglab.janelia.org/proj/mRMR/, and run with the parameters: λ=1, m = MID.

### (2) Incremental Feature Selection (IFS)

As mentioned above, the feature components could be ranked using mRMR method. But it's not uncovered that which components of the feature would be most necessary. The IFS method was adopted in this study to perform feature selection for analyzing the key properties related to allergenicity. Based on the ranked features obtained from the mRMR, 128 feature sets were constructed by adding one component to the set at a time in the order of mRMR features list. The  i -th set is formed like $S_{i}^{{}^{\prime }}=\left\{f_{1}^{{}^{\prime }},f_{2}^{{}^{\prime }},\cdots \;,f_{i}^{{}^{\prime }}\right\}\left(1\leq i\leq 128\right)$, where $f_{i}^{{}^{\prime }}$ means the feature at the  i -th position after ranking by mRMR.

For each of feature sets, an SVM predictor was constructed and its ten-fold cross-validation performance was derived. Eventually, an IFS curve was obtained, with the component number  i as its *X*-axis and the corresponding sensitivity, specificity and accuracy as its *Y*-axis. If the IFS curve has a inflection point at *X*=*h*, the feature set that played a key role in allergenicity would be $S_{optimal}=\left\{f_{1}^{{}^{\prime }},f_{2}^{{}^{\prime }},\cdots \;,f_{h}^{{}^{\prime }}\right\}$.

### Ten fold cross-validation

The performances of all methods applied in this study were evaluated using ten-fold cross-validation. The dataset was randomly partitioned into ten subsets, where each subset has nearly equal number of allergens and non-allergens (negative controls). Of the ten subsets, a single set was retained as the validation data for testing the method, and the remaining nine subsets were used as training data. This process was then repeated 10 times with each of the ten subsets used exactly once as the validation data. The overall performance of a method was the average performance over ten subsets.

## Results

### Model construction with IFS

**Figure 1:**
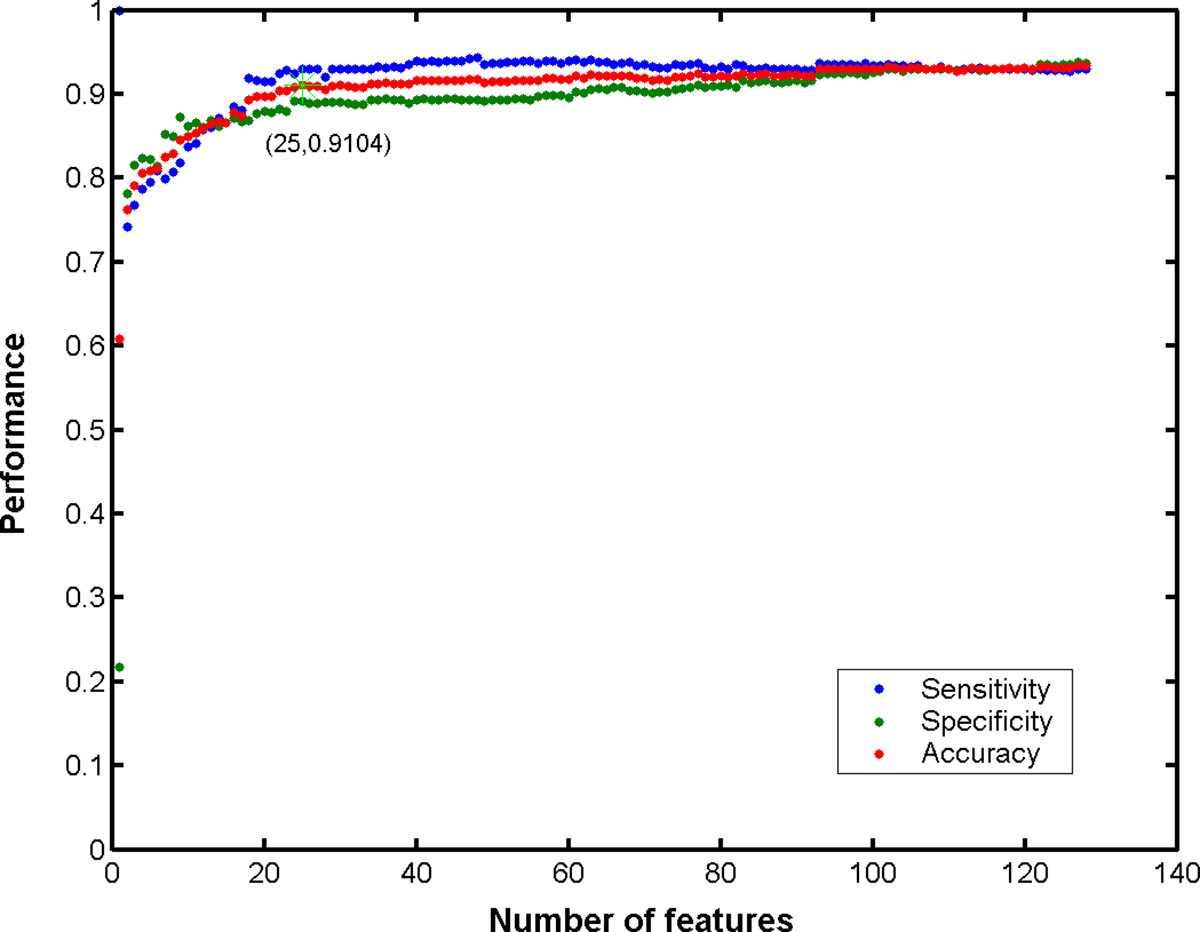
**IFS curves of all proteins in training dataset**. IFS curves of 128-D feature space. The overall accuracy reached its inflection point of 91.03% at the number of feature components used was 25.

As described in the method section, 128 feature sets were built, and the corresponding prediction models were then constructed and evaluated. As shown in Figure 1, it reached the inflection point of IFS curve at accuracy of 91.03% when the number of feature components used was 25. In other words, these 25 feature components selected by mRMR would compose the critical feature set for the classifier of allergen/non-allergen. We analyzed the 25 feature components in the next section to understand key factors for protein's allergenicity.

### Optimization of feature components

**Figure 2:**
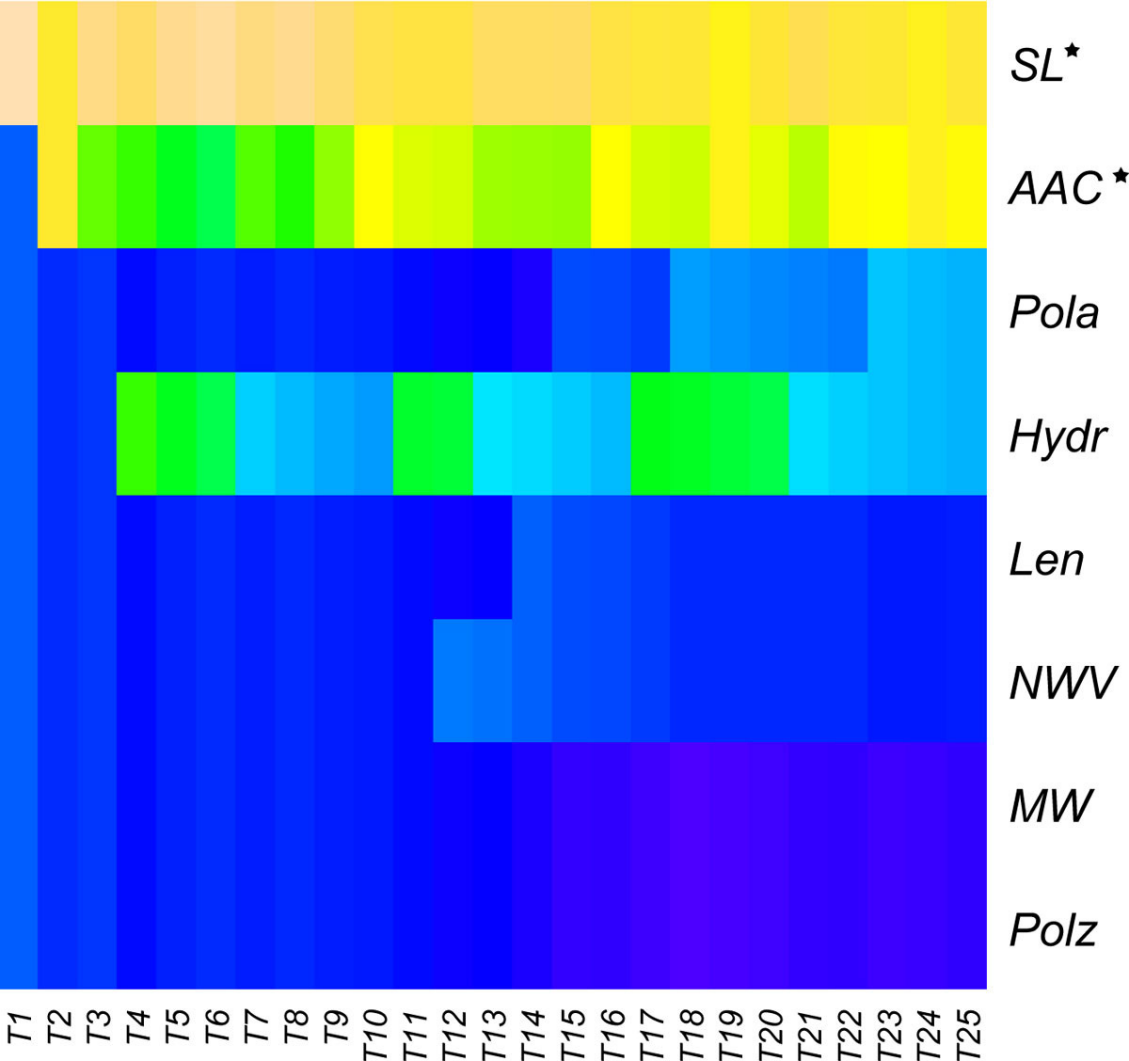
**The heatmap of feature compositions of different property types**. The vertical axis represents the feature composition of eight types of protein properties, i.e. SL (subcellular locations), AAC (amino acid composition), Pola (polarity), Hydr (hydrophobicity), Len (length), NWV (normalized van der Waals volume), MW (molecular weight) and Polz (polarizability). While horizontal axis shows the selected numbers of top features in IFS procedure signed as *Tx*, in which *x *denotes the number features. And the warmer colour denotes the higher correlation. The properties with star (SL* and AAC*) performed remarkable.

To investigate which features are crucial for protein's allergenicity, we extracted the 25 feature components at the inflection point from mRMR list, in which two of five property types, "subcellular locations" and "amino acid composition", were significantly enriched by hypergeometric test (p-value < 0.05, Benjamini-Hochberg correction) (Table 2). A heatmap in Figure 2 also illustrated that the features of AAC and SL (subcellular locations) were remarkable [50]. We further try to figure out which of the 22 subcellular locations of particular importance in allergen prediction by taking look at the SL distribution in soybean (*Glycine max*) and wheat (*Triticum aestivum*). So far, these two species had most known allergenic proteins. The results revealed that endoplasmic reticulum for soybean only and other two SL (extracellular/cell surface and vacuole) for both soybean and wheat were significantly more enriched in allergens compared to randomly selected proteins (p-value < 0.05) (Table 3 and Additional file 2).

**Table 2:** The optimal feature components

	SL	AAC	Hyd	Pola	NW
**PN^a^**	22	20	21	21	21
**SN^b^**	9	8	3	3	1
**p-value**	0.0365	0.0345	0.2052	0.2052	0.06983

^a ^PN means successes number in population; ^b ^SN means successes number in sample.

**Table 3:** The subcellular location analysis

Corrected p-value	End-ret^1^	Extr-sur^2^	Vacuole
*Glycine max*	0.0003	0.0210	3.8E-9
*Triticum aestivum*	--	0.0036	0.0314

^1^End-ret means Endoplasmic reticulum; ^2^Extr-sur means Extracellular +cell surface.

### Allergen predicting by category

**Figure 3:**
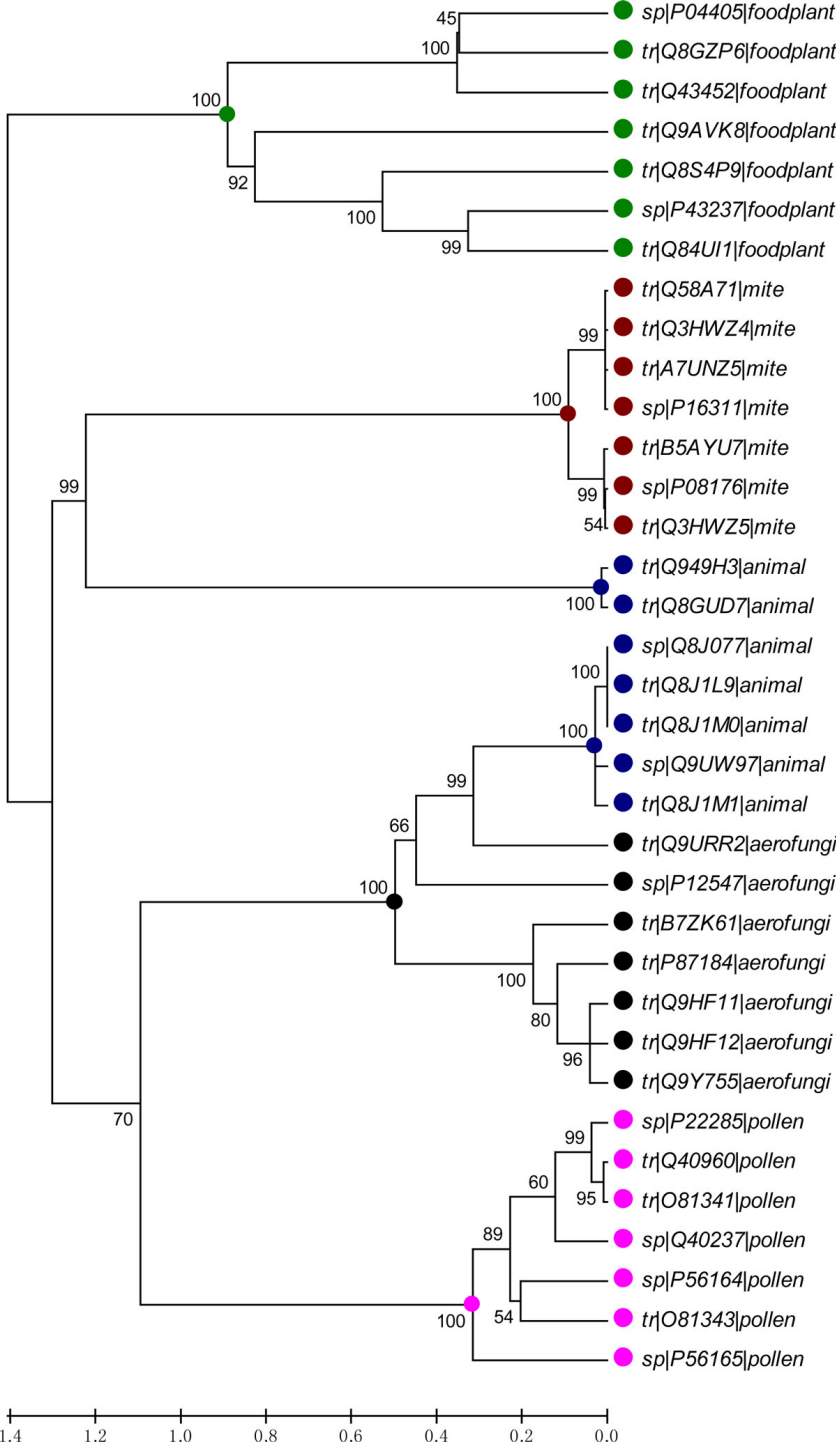
**The NJ tree of 35 allergen sequences from five categories**. The topology of this tree was generated using MEGA 5, summarizing the evolutionary relationships among the allergens from different categories. The branches of the same category were colour-coded.

**Figure 4:**
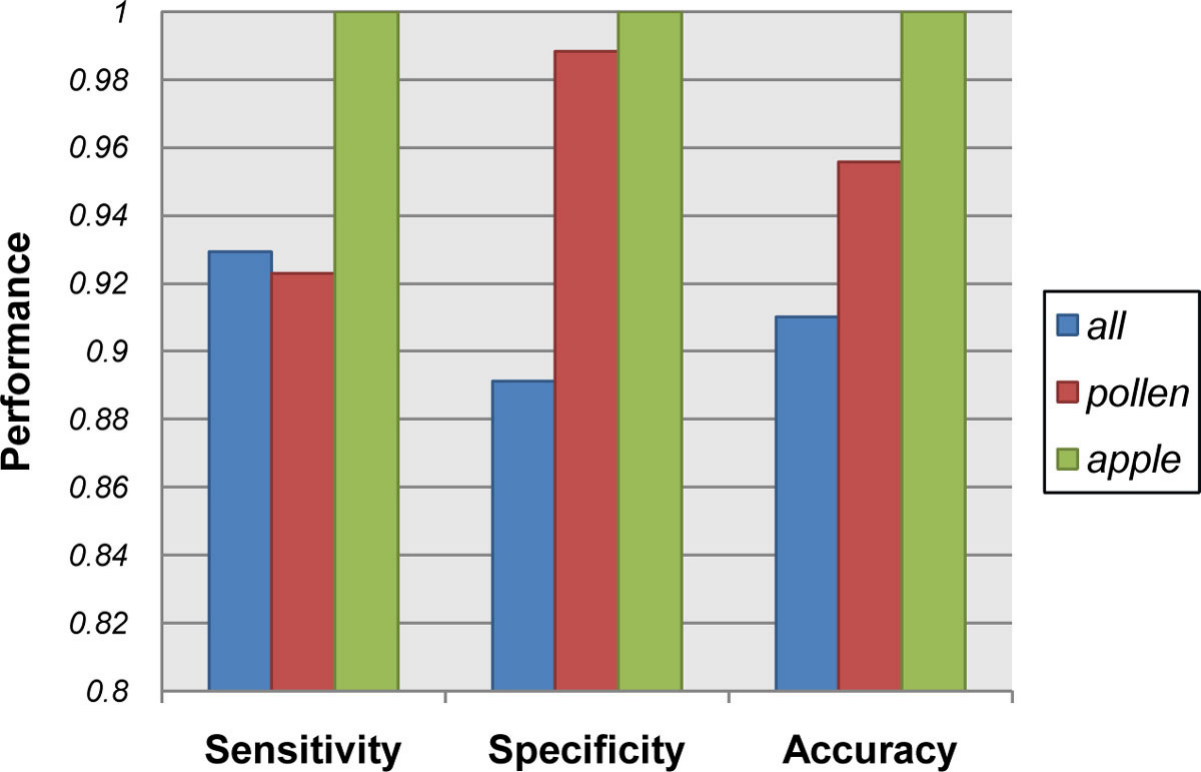
**Performance comparison in Pollen, Apple and all known allergens**. The chart illustrates the performance comparison of predictors based on 128-D feature vector models within Pollen and Apple against within all known allergens.

Since people who concern about allergenicity usually focus more on a specific species or category like food-plant rather than all species, we performed a multi-alignment and constructed a phylogeny tree using MEGA software (version 5.0) [37] for 116 allergens which sequence length is between 240 and 600, from the biggest two sub-families in six major categories (Aero-Fungi, Animal, Apple, Food-Plant, Mite and Pollen) respectively. 909 allergens were included in these six major categories, which account for over 77% of all allergens. The NJ (Neighbour-Joining) tree (Figure 3, Additional file 3) illustrated that the sequences of allergens were more conservative within category than between categories. Hence, we attempted to build and evaluate our predictor within Aero-Fungi, Animal, Apple, Food-Plant, Mite and Pollen individually. As displayed in Figure 4, the category-specific models in Pollen and Apple outperformed full model. Even the accuracy of allergen prediction in Apple can reach 100%.

### Comparison with existing methods

**Figure 5:**
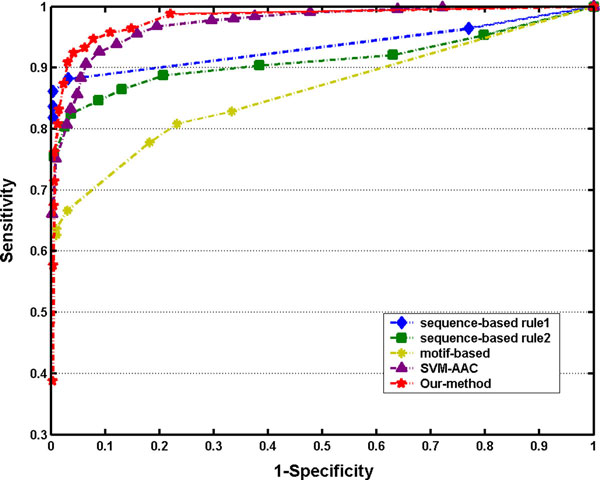
**The ROC curves of various approaches for allergen prediction**.

We compared the performance of our method with the existing approaches for allergen prediction. So far there are three major kinds of computational methods for allergen prediction including FAO/WHO criteria, motif-based method and SVM-based method. Among the SVM-based methods, SVM-AAC taking the amino acid composition as feature vectors is mostly common used. The ROC curves illustrated the superiority of our 128-D feature vector models to the others, in which the overall accuracy reached its peak of 93.42% (Figure 5).

### Web-based application

**Figure 6:**
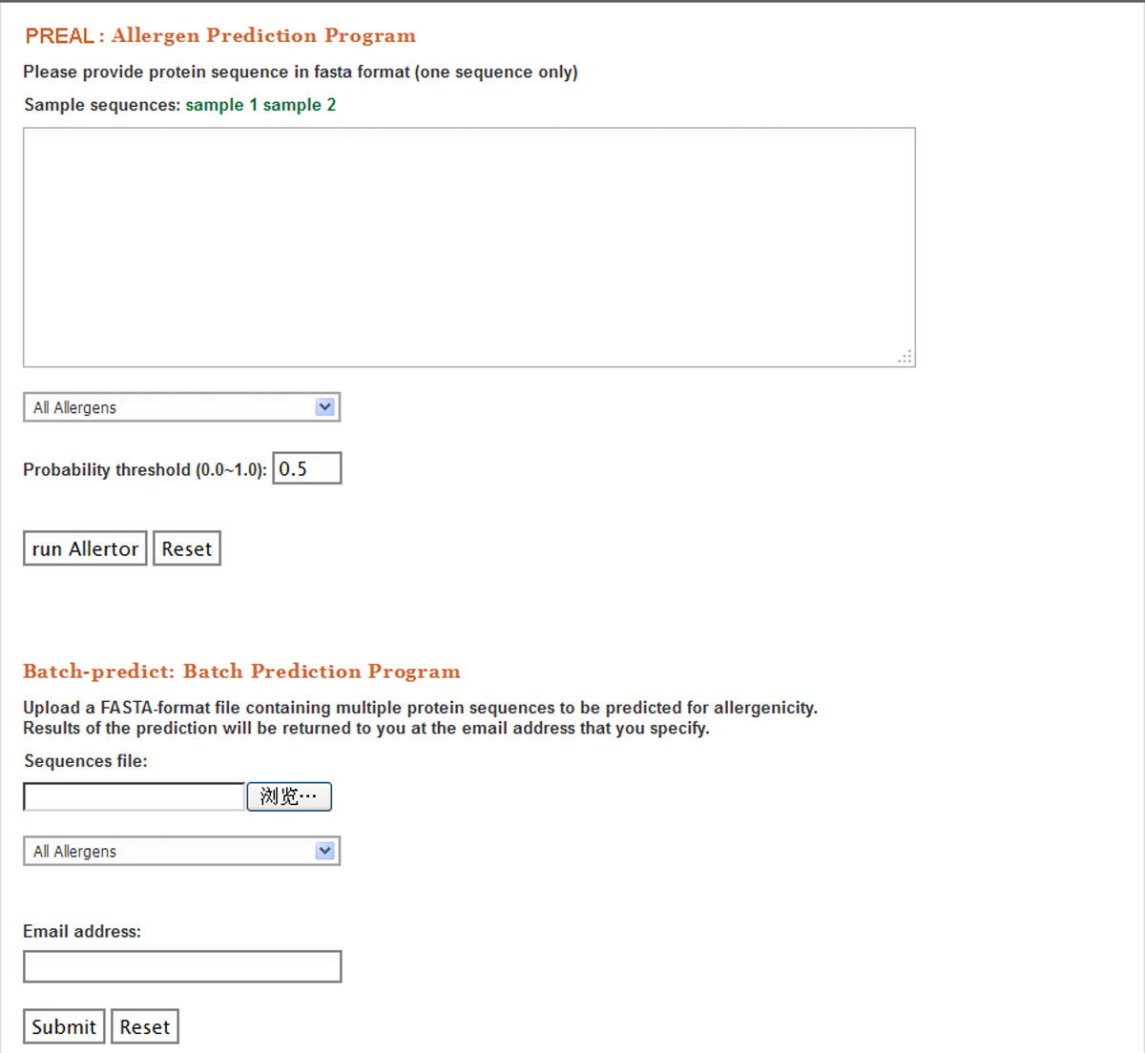
**A snapshot of the prediction page of the web application**.

A web server named PREAL (http://gmobl.sjtu.edu.cn/PREAL/index.php) has been developed that allows people evaluate the potential allergenicity of protein(s) on-line using our new method. When a query protein sequence in FASTA format is given, PREAL will report the putative allergenicity. Besides, both category-specific and full model are available in PREAL. PREAL also provides batch prediction, which returns the results by E-mail. A snapshot of the prediction page of PREAL was displayed in Figure 6.

## Discussion and conclusions

The aim of this study is to predict the potential allergenicity of proteins efficiently and analyze the key factors resulted in allergenicity. We developed a new SVM-based model by integrating various biochemical and physicochemical properties, as well as sequential features and subcellular locations. The ten-fold cross-validation indicated that the predictor can achieve from 93.42% to 100% overall accuracy. Considering the secondary structure propensity and solvent accessibility contribute to the protein's stability and function, we also expanded our model by adding these two kinds of property. As predicted by SSpro [31], an amino acid can be grouped as: helix, strand or coil for the secondary structure propensity (SSP), and the solvent accessibility can be classified into buried or exposed to solvent predicted by ACCpro (Table 1) [32]. Finally the model can be formulated as a vector in a 156-D (dimensional) space. But the corresponding evaluation indicated the overall accuracy could be increased only 0.01 by the 156 features model while its running time was more than 60 times longer than the 128-D model.

With the feature selection procedure based on the mRMR and IFS methods, we found that the subcellular locations and amino acids composition would play the crucial roles in determining the allergenicity of a protein. For soybean and wheat, the extracellular/cell surface and vacuole are observed to be the exactly effective locations. Key effect factors for allergenicity have not been reported before. Because allergenic proteins had higher sequence similarities within categories, we also carried out the predictor in six major sub-sets in which higher accuracy was obtained. To facilitate application, we built a web-based application providing the prediction approach presented in this paper on-line, so that people can perform a test even large-scale testing expediently.

Despite this, there are some issues should be addressed in further the study. Although the allergen prediction within category preformed pretty well, small amount of allergenic proteins were captured within some category limited its wide usage. Another issue is the difficulty in effective validation of a new method presented by wet experiments expect for the cross-validation.

## List of abbreviations used

mRMR: Maximum Relevance Minimum Redundancy method; IFS: Incremental Feature Selection; GO: Gene Ontology; WHO: World Health Organization; FAO: Food and Agriculture Organization; SVM: support vector machine; ARP: Allergen Representative Peptides; AAC: amino acid composition; MW: molecular weight; SSP: secondary structure propensity; NWV: normalized van der Waals volume; NJ: neighbour joining.

## Competing interests

The authors declare that they have no competing interests.

## Authors' contributions

JW carried out the programming and analysis studies, and drafted the manuscript. JL conceived of the study, and participated in the manuscript draft. DBZ supervised the research. All authors read and approved the final manuscript.

## Supplementary Material

Additional file 1 **The 128 features for allergen protein identification**. Click here for file

Additional file 2 **The statistical data of subcellular locations for soybean and wheat**. There are 22 subcellular locations (SL) for eukaryotic proteins. Only SL terms located by 3 more allergens were calculated. Click here for file

Additional file 3 **The NJ tree of 116 allergen sequences from six categories**. The topology of this tree was generated using MEGA 5, summarizing the evolutionary relationships among the allergens from different categories. The branches of the same category were color-coded. The NJ tree was consisted of 116 allergen proteins which met the condition of sequence length is between 240 and 600, and protein family accounted for a higher proportion within the categories. Click here for file

## Declarations

The publication costs for this article were funded by National Transgenic Plant Special Fund of China (2011ZX08011-006).

This article has been published as part of *BMC Systems Biology *Volume 7 Supplement 5, 2013: Selected articles from the International Conference on Intelligent Biology and Medicine (ICIBM 2013): Systems Biology. The full contents of the supplement are available online at http://www.biomedcentral.com/bmcsystbiol/supplements/7/S5.
