# Supplementary figures and images for: PREAL: prediction of allergenic protein by maximum Relevance Minimum Redundancy (mRMR) feature selection
Source: BMC Syst Biol. 2013 Dec 9;7(Suppl 5):S9. doi: 10.1186/1752-0509-7-S5-S9 (PMC4029432; doi:10.1186/1752-0509-7-S5-S9)

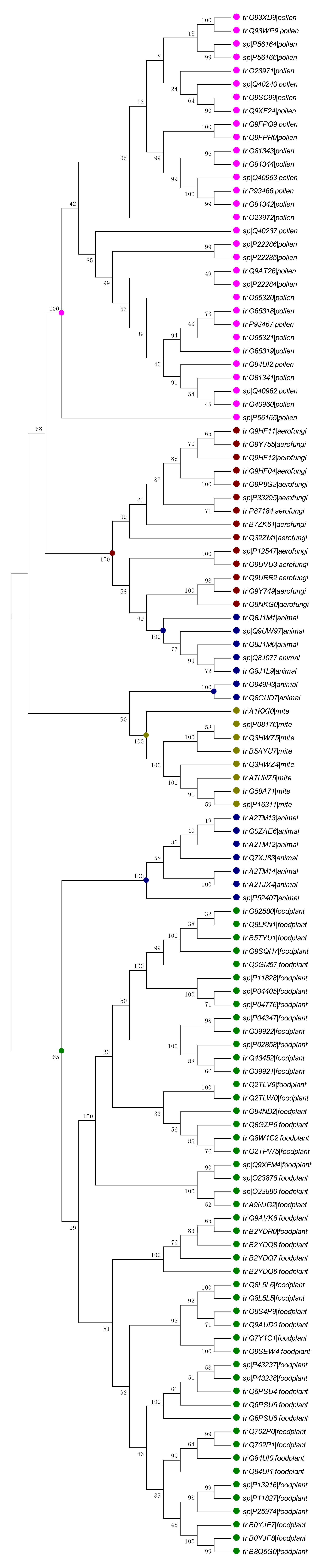

Supplement: Additional file 3 — The NJ tree of 116 allergen sequences from six categories. The topology of this tree was generated using MEGA 5, summarizing the evolutionary relationships among the allergens from different categories. The branches of the same category were color-coded. The NJ tree was consisted of 116 allergen proteins which met the condition of sequence length is between 240 and 600, and protein family accounted for a higher proportion within the categories. [file 1752-0509-7-S5-S9-S3.tif]
